# Mental Health Collaborative Care in Brazil and the Economy of Attention: Disclosing Barriers and Therapeutic Negotiations

**DOI:** 10.1007/s11013-024-09852-w

**Published:** 2024-04-23

**Authors:** Manuela Rodrigues Müller, Francisco Ortega

**Affiliations:** 1 Faculdade de Ciências Médicas, Universidade do Estado do Rio de Janeiro (FCM-UERJ), Rio de Janeiro, Brazil; 2 Catalan Institution for Research and Advanced Studies (ICREA), Barcelona, Spain; 3 Medical Anthropology Research Center, Universitat Rovira i Virgili, Av. Catalunya 35, 43002 Tarragona, Spain

**Keywords:** Mental health collaborative care, Primary health care, Therapeutic negotiations, Economy of attention, Brazil

## Abstract

The introduction of mental health collaborative care (MHCC) is one of the strategies to scale up access to mental health care in primary health care in Brazil. This article investigates an experience of mental health collaborative care in the city of Rio de Janeiro, Brazil. It is a qualitative study involving interviews with physicians and mental health professionals working in primary health care units located in the northern part of the city of Rio de Janeiro, Brazil. The aim is to examine the various strategies and negotiations that primary health care professionals deploy to identify mental distress and plan health care interventions. We discuss the results within the economy of attention framework. We argue that divergences in diagnostic design and therapeutic planning carried out by professionals and users or observed in MHCC meetings illustrate the health-disease-care seeking phenomenon as a negotiated process, entangled in complex interactions. Our results evince that those interactions are not always evident and configure 'what is at stake' in mental suffering. The incorporation of cultural and structural determinants in collaborative care may enable the expansion of mental health initiatives sensitive to local needs and realities.

## Introduction

Brazil has a well-structured National Health System (Sistema Único de Saúde or SUS) with nearly universal access to health care services. The SUS developed in the late 1980s as part of the ‘Health Reform Movement’ (Movimento da Reforma Sanitária) against the military dictatorship and played a key role in the process of redemocratization in the country (Massuda et al., 2018). The reorganization of Brazilian public healthcare has been oriented towards the structuring of primary health care following Alma Ata’s recommendations.

The Family Health Strategy (FHS) is the Brazilian model of primary health care. The FHS qualifies care practices for families and communities by identifying needs and organizing interventions in the territories under its responsibility (Alencar et al., 2014). FHS teams include family physicians, nurses, and community health workers (CHW) (Alencar et al., 2014; Almeida, 2018; Schneider et al., 2020). Each team has a sanitary responsibility for a territory which encompasses between 2000 and 3500 people. Guidelines for organizing the teams give preference to hiring family physicians with specific training in family and community medicine (Portaria nº 2.436, 2017). Since there are not enough family physicians available to be hired and many physicians do not want to work in vulnerable parts of the city, several teams must rely on general practitioners, or physicians with other specialties, without the necessary training to work at this level of care (Almeida, 2018; Müller, 2019). The introduction of community health workers within FHS has produced successful experiences of lay health care agents mediating health services and the population, ensuring greater cultural sensitivity in the interventions developed in the community (Cardoso & Nascimento, 2010; Morosini & Fonseca, 2018; Nunes et al., 2002). PHC services are part of a regionalized health service network comprising specialized services, including mental health services designed in accordance with the basic tenets of the Brazilian psychiatric reform.

Brazilian psychiatric reform began in the late 1970s, and it was influenced by Latin American social medicine and more specific Brazilian collective health and by psychiatric reform movements in other countries such as Italy, France, and England (Nunes et al., 2007; Yasui et al., 2016). For the Brazilian reform, deinstitutionalizing goes well beyond dehospitalization. It involves new forms of care and social insertion that deal with the sociocultural, epistemological, and legal-political dimensions of mental suffering (Yasui et al., 2016). The wider perspective resulted in the so-called “psychosocial care” (*atenção psicossocial*) paradigm and a community-based care model involving the creation of community mental health care centers (CAPS or psychosocial care centers). As a result, care practices and mental health services are part of the healthcare network of a catchment area. The sociocultural diversity of each community is a fundamental aspect for adequate care and for overcoming exclusionary and stigmatizing practices (Amarante & Nunes, 2018; Yasui et al., 2016).

Mental health interventions in PHC were introduced in the 2000s with the evolution of the implementation of the Family Health Strategy in the country. *Matriciamiento*, or *matrix support*, is the Brazilian term for mental health collaborative care (MHCC) in primary health care. It is a strategy to qualify mental health care within FHS. It provides specialized technical support offered to an interdisciplinary health team in order to expand its field of action and qualify its interventions. It draws on shared work, co-management, intersectorial care planning, interdisciplinarity, and a broad view of the health-disease-care process, in its social, health, and pedagogical dimensions. It aims to build co-responsibility in health care, the expansion of the scenarios in which specialized care is provided, and the shared construction of clinical and health guidelines between the health team and the specialists who offer MHCC (Campos et al., 2013; Chazan et al., 2020; Chiaverini et al., 2011; Cunha & Campos, 2011; Santos et al., 2017).

MHCC is provided by CAPS and family health support centers (*Núcleos de apoio à saúde da família*, NASF). NASFs include multiprofessional teams and have been the main source of MHCC since 2008 (Santos et al., 2017). Central to MHCC in the country is the restructuring of work relations between professionals and levels of care. Hence, the emphasis given to the political aspect of mental health care and the democratization of relations between professionals, resulted in a direct involvement of mental health professionals in the co-management of cases (Ortega & Müller, 2022; Saraiva et al., 2020).

The collaborative care process was greatly stimulated by the Ministry of Health, which regulated the actions and produced guidelines for implementing this strategy across the municipalities (Chiaverini et al., 2011; Santos et al., 2017). However, in recent years, there has been a progressive weakening and dismantling of the SUS and mental health policies in the country — including the financial support of Mental health collaborative care, associated with the advancement of neoliberal policies which heavily impacts health care provision (Almeida, 2018, 2019; Mendes, 2016).

This article examines an experience of mental health collaborative care in the city of Rio de Janeiro as an illustration of mental health interventions in local contexts. We conducted semi-structured interviews with family health physicians and mental health professionals working with MHCC. Our goal was to analyze the diverse strategies developed by primary health care professionals to identify and treat mental suffering and plan care interventions in the context of MHCC. The experience of mental health collaborative care examined in this article can enrich the debate on expanding mental health initiatives that are sensitive to local needs and realities, as practitioners' training and experience in recognizing mental health cases and planning follow-up strategies are central to the understanding of health care as determined by social pathways. Structural and sociocultural issues affect each region and team differently, and health teams and healthcare managers need to identify and integrate these variations in the planning of health interventions.

The economy of attention was an useful analytical framework to elucidate professionals’ experiences with mental health issues. Adams et al. (2019) proposed the notion of “economy of attention” to highlight that health problems are prioritized, or neglected, according to practitioners' and managers' understanding of what constitutes a health problem. The “social apparatus of care” formed from the interactions of social actors involved in a given scenario configures a complex intertwining of material and human conditions related to the health-disease phenomenon. Health problems are addressed or not according to the social interactions that shape them. A mechanical understanding of the social and the structural determinants of health without an in-depth exploration of the different forms these determinants influence care practices ends up limiting the impact of health interventions. Many causal social pathways are involved in health care, and it is useful to “focus on interconnections between health disease causation and larger social, biological, political and economic contexts that cause harms” (Adams et al., 2019, p. 1390).

Professionals’ narratives evince that the interactions of different actors—users, general practitioners, family health physicians, mental health professionals—in local care contexts articulate complex epistemologies of care and involve negotiations of problems, priorities, and ways of caring. We call these articulations therapeutic negotiations and argue that the experience of collaborative care can be very powerful in exposing such negotiations and the role of social actors in these exchanges, as well as to better understand aspects related to access and quality of care. The idea of negotiation should be understood more broadly than a direct transaction—as if doctor and patient were in a transaction to decide on an intervention. Sensitivity to the sociocultural elements of the health-disease phenomenon allows professionals to deepen approaches and diversify offers. In this sense, negotiation involves a sensitivity to social determinants, or social determination, implying a broader understanding of the health-disease phenomenon and recognizing users as agents of care practices.

The illustrations of cases and therapeutic negotiations highlight actors’ intentionality and their ability to appropriate the diverse structures (neighborhood, economic-political, technical-scientific) that shape their actions. In this sense, care practices, objects, and therapeutic itineraries chosen by people serve as indicators of broader social processes (Menéndez, 2003; Müller et al., 2022). Individuals and social groups promote and articulate diverse forms of care in a pragmatic attitude towards their health problems aiming at ensuring the biosocial reproduction process (Menéndez, 2003, 2016).

Understanding the health-disease-care seeking phenomenon as a negotiated process is not always evident. The divergences and therapeutic negotiations taking place in the daily life of health services may expose “what is at stake” in mental suffering (Kleinman, 2006).

## Methodology

We carried out qualitative research involving semi-structured interviews with FHS and MHCC professionals at two health units in the city of Rio de Janeiro, Brazil. These units were located in the northern part of the city and belong to the Family Health Strategy and are identified as Family Clinics (FC). It is an area that diverges from the more affluent parts of the city, and it is formed by 17 neighborhoods, some very violent due to drug trafficking and police raids. This region comprises 25 health units, responsible for 444,961 users (CAP 3.2, n/d). We selected two units for our fieldwork, identified by the letters A and B. Both are family clinics, have six family health teams each, and are responsible for 14,133 users (A) and 14,713 users (B). These units were chosen based on convenience, considering their accessibility by public transport and less violent localization, so we were able to visit the units and interview the health professionals safely.

Before starting the individual interviews, we presented the project at the health units team meetings and invited the professionals to participate. Manuela Rodrigues Muller (MRM) only took part in these two team meetings and then returned to the units to interview the professionals. We chose to interview professionals directly involved in diagnostic construction and therapeutic planning in mental health (general practitioners, family health physicians, and mental health professionals), who met the inclusion criteria, which was to work for more than 6 months in family health strategy. 11 professionals were interviewed: 3 family health physicians, 5 general practitioners, and 4 mental health professionals (2 psychologists, a social worker and a physical educator, all with multiprofessional residencies in mental health). Most of the interviewees were women (8 out of 11), with an average age of 30. The professionals interviewed in each unit were given pseudonyms in order to preserve their identities.

In Brazil, mental health collaborative care can be provided by psychiatrists, psychologists, social workers, nurses, and other professionals with expertise in mental health. There are multiprofessional residency programs with an emphasis in mental health care whose graduates may work in mental health collaborative care and/or mental health services. The teams selected for the interviews were not followed by psychiatrists, because, as in other regions of the city, there are difficulties in hiring those professionals. This is due to the low salaries, absence of a career plan, and fragile working conditions involving working in violent parts of the city (Müller, 2019; Goulart et al., 2021).

Episodic narrative interviews (semi-structured interviews) with general practitioners, family health physicians, and mental health professionals were conducted in person by Manuela Rodrigues Muller (MRM), at the interviewees’ workplaces after previous scheduling. The interviews lasted between 30 minutes and 1 hour. The interviews were recorded on audio and transcribed with the consent of each interviewee and respecting the ethical principles of health research. All data collection received clearance by the Ethics Committees of our institution – (Clearance code X1) and the Municipal Health Secretariat of Rio de Janeiro (Clearance code X2), and all participants provided informed consent.

Interviews focused on practitioners’ experience in managing the mental health cases they identified and the role of collaborative care in these situations. Thus, professionals were asked to describe how they planned therapeutic interventions for the cases followed by the health teams, taking into account their participation in MHCC.

We did not conduct interviews with CHWs because they are not directly involved in elaborating users’ therapeutic plans, but many of the interviewees mentioned their experiences with the CHWs, indicating the importance of their work for the health teams and the need for more discussion and reflection on their influence on healthcare within FHS (Morosini & Fonseca, 2018; Nunes et al, 2002).

The structure of the interviews follows the Episodic narrative interview. This format allows the interviewees to present their experiences contextually. The episodes (here, the case reports) are seen as the object of these narratives and as a way of accessing relevant experiences of the subjects. Routines and everyday events can be analyzed without the interviewees feeling constrained by the theoretical domain of the theme (Flick, 2000). Besides, professionals' reports about the people they follow, or situations experienced in their daily care, end up revealing unconscious assumptions and conflicts, since they are not aware of their prejudices, values, and criteria when it comes to prioritizing some decision over others (Müller, 2019).

We use Discourse Analysis (Flick, 2014) to analyze the interviewees' statements. This methodology seeks to investigate subjects' specific linguistic choices and what they represent in a given social context. The use of language both expresses and shapes certain worldviews. In this sense, discourse analysis goes beyond speeches’ literal sense to access their specific meanings (according to the speaker’s positionality) and reflects on how those speeches interact with the context in which they are issued (Orlandi, 2003, 2011).

Mental health case reports and their discussions at mental health collaborative care meetings provided the material for the interviews and a field diary helped the interpretation process. The diary was kept by Manuela Rodrigues Muller (MRM); the observations made during the visits to the services about the environment in the units and the way the interviews took place were noted down. The diary was used to contextualize the fieldwork, compare findings, and enrich the analysis of the interviewees' narratives. Discursive formations and conceptual axes were created based on an in-depth reading of the material, supported by the field diary, allowing the significant nuclei of the statements to be identified and its meanings to be interpreted taking into account the speakers’ positionality and the contexts in which the interviews took place (Orlandi, 2003, 2011).

## Negotiating Care Epistemologies and Therapeutic Interventions in Family Health Strategy

We examine in this article the various strategies and negotiations that primary health care professionals deploy to identify and deal with mental distress in the context of mental health collaborative care. The empirical material selected for this article refers specifically to the guiding question “how do practitioners identify mental health cases?” It became evident during the interviews conducted with general practitioners, family physicians, and mental health professionals around their mental health collaborative care practice that the central issues for them were how family health professionals recognize mental health cases and how they plan follow-up strategies. The professionals we interviewed gave detailed and distressed reports. They were concerned with the difficulty in offering quality health care to users and their families as they stressed larger determinants (such as work processes, interprofessional relationships, health service network, and sociocultural factors) impacting their interventions.

The excerpts presented in this article were selected after analyzing the significant nuclei and noting that they referred to the following dimensions: team composition and work processes organization; sociocultural characteristics of the territory and availability of resources in the community; and the broader sociopolitical structure that configures the health system in the country. These dimensions emerged when reading the material as we noticed that professionals described the clinical situations taking into account different elements: actors (patients, family members, neighborhood; other professionals: CHW and managers); interactions (in individual meetings, activities with groups, institutional relations, contact with the community in home visits, neighborhood association meetings, neighborhood parties, vaccination campaigns); and scenarios (within the health services, in the territory attached to the health unit, in other services). Thus, we realize that the interaction of these elements shapes the approaches to cases, revealing the contingent nature of care priorities in a given scenario highlighted by the economy of attention. When we examine the professionals’ experience, we observe in a situated way the social processes and actors involved in shaping mental health care in PHC in Rio de Janeiro.

In addition, the juxtaposition of excerpts from different interviewees gives visibility to the unique aspects observed in the communities and health units where the professionals work, even though health care is guided by the same principles (of the SUS) and managed in the same way by the municipal health department. The speeches reveal the interweaving of the dimensions, the balance of different elements, and the “economy of attention” influencing each case. Below we present the three dimensions identified in the interviews and how they appeared throughout the professionals’ narratives.

Regarding team composition and work processes organization, some excerpts are presented below. Our interviews with physicians with and without training in family medicine confirmed what Pinto (2017) already noted., i.e., that family health professionals conceive mental health cases in a broad way, influenced by their professional training, expertise in primary health care and insertion in the community. General practitioners tend to resort to biomedical definitions (perhaps due to their recent insertion in PHC and their initial biomedical background) and family health physicians and community health workers tend to use more relational understandings, drawing on their knowledge of users and their families.

I had a patient who yelled at me, but it was a matter of prescriptions. (...) It was the only problem I have had with a psychiatric patient. (...) There are many people that I see that are a little more educated, so it is easy for you to deal with them, to explain. There are some that arrive and don't understand anything. They don't know what the medication is for, or anything. (Alice, general practitioner)

Two users, both with schizophrenia, who lived with an elderly aunt and had virtually no financial resources. Only one had worked and was now retired due to illness. The other user fell ill very early in his life and did not work. We tried to get him some sort of social benefit, but we failed. We helped with what we could: medication, inclusion in the physical activities at the unit. Then, we arranged, through his community health worker, who is part of a church group, to get the user’s house cleaned, painted, tidied up. So, we raised some money and provided them with food (Antonia, family health physician)

Alice is a newly graduated physician, with little experience in family health. Her report evinces difficulty in understanding the patient's impatience. It is quite frequent that patients and doctors have relational problems at the health unit due to the extreme amount of consultations doctors in primary care must face and also to the profound class differences and social inequalities that exist between health professionals and patients in Brazil. In the situation described by Alice, she identifies the patient's low level of education with his difficulty in accepting the prescription, limiting the inclusion of other elements that make the patient's demand legitimate or understandable.

Conversely, Antonia is a physician who has worked at the unit for about 20 years and has participated in implementing the family health strategy there. She acknowledges the patient’s psychiatric condition, but she stresses that her biggest problems are the family's socioeconomic conditions. Besides conventional health interventions, such as prescription of medication and physical activity, the members of her team sought to solve problems related to income and feeding. In this sense, when comparing the two approaches, an important difference stands out. Antonia and her team locate the family's suffering within a psychosocial framework, seeking to create networks of cooperation mediated by the community health worker and including the neighborhood. Despite the difficulty in establishing consensus on the nature of the affliction and intervention priorities, this team could translate this entanglement of needs into care (Chase, 2021).

Furthermore, these two care experiences expose different understandings about the social determination of health and illness. While Alice takes education literally to justify the assertive behavior of the patient, Antonia includes the family's socioeconomic difficulties in explaining the gravity of the situation and planning the intervention.

Case discussions at medical schools focus on the description of the patient’s health problems. However, some respondents gave more nuanced responses. This was more often with mental health professionals, while physicians remained more focused on individual cases given their training largely focused on individual, ambulatory or ward care. In a certain sense, it seems only natural that physicians are attentive to individual situations, observed in routine consultations, while mental health professionals with greater circulation in the territory may expand their understandings and reports and provide broader views on users' mental health status and therapeutic resources. This greater circulation is facilitated through home visits along with community health workers, but also through group therapy at the health unit and collaborative care meetings in different settings, as mental health professionals usually supervise more than one health unit and its teams.

A male patient is quitting treatment, so we are planning a home visit with someone he trusts from CAPS to have a better understanding of his reasons. You see, when he first arrived at the health unit, he was in such a state he wanted to “give his legs away” [a psychotic symptom], then he was hospitalized. Afterwards, he was discharged with a long-term prescription which he did not renew. His mother plays along. She thinks her son is doing very well, that he doesn't need treatment. It's like the mother has prejudices regarding mental disorders and fears that her son will be diagnosed, so she states that her son is not crazy. “My son is working. You see, he does not need this medication”. (Beatriz, general practitioner)

Angolans. The suffering of these people is very much related to the issue of religion ... And with many issues related to skin color, related to their culture, our culture that is different from theirs. I say “look, we can think about it together over time”. I started to make this point at the mental health collaborative care meetings. I asked the family health team if they had noticed the difference in the way of taking care of their health and that we needed to start thinking about it to promote specific care for that family. (...) I think that from this sensitivity, together, we start to accommodate a growing demand. (André, mental health professional)

Beatriz is a physician at Unit B. She interacts well with the family health team and participates in mental health collaborative care meetings. Despite assisting the patient's whole family and including other professionals from CAPS in care planning, she does not realize that the young man's care should also involve caring for his family members, listening attentively to them, specially tuned to their understanding of mental disorders. Including cultural beliefs, practices and social norms are a challenge to scaling up mental health care into primary health care and ought to be addressed (Faregh et al, 2019), but professionals frequently stick to individual approaches.

In contrast, André, a mental health professional and a supervisor at Unit A, introduced the difficulties of Angolan families newly arrived in Brazil at MHCC meetings, in which strategies to provide greater attention to these families and an examination of their living conditions and cultural background were discussed.

Such clippings may be used to illustrate the effects of training and experience in family health but also of work processes in the follow-up of families. Family physicians and mental health professionals tend to be more aware of and incorporate sociocultural aspects in the planning of interventions. The attention to the sociocultural determinants involved underlying Andre’s observation is central for family health care planning. The inclusion of other elements in clinical reasoning, such as beliefs and values or the experience of discrimination, beyond the conventional identification of psychiatric symptoms makes it possible to incorporate the local idioms of distress of patients and their family members and to expand care initiatives in a negotiated way, taking into account the care provided in the community. Moreover, it reveals the difficulties regarding the understanding of belonging to a social milieu and the unfoldings of this understanding in health practices. Social “belonging” is configured by tensions and the analysis of these divergences “benefits from a more flexible, intersectional, and contingent reframing of the social” (Adams et al., 2019, p. 1385), as Antonia’s and André’s testimonies reveal.

In Brazil, different from other contexts (Lie & Greene, 2021), guidelines in primary health care promote the construction of intersectoral actions (Chiaverini et al., 2011; Brazil, 2017) and some health professionals recognize that users’ complaints and demands are related to situations of social vulnerability and to characteristics of the territory, such as unemployment, loss of income, migration, domestic, or community violence. But the articulation of family health teams with other sectors, such as social care and legal assistance, is not always adequate due to institutional, structural, and even mobility difficulties in the communities, which ends up reducing health interventions to drug prescription or psychological care.

As displayed in the following excerpts, interviewees' discuss difficulties in delivering health care associated to the availability of resources in the community and difficulties in coordinating care strategies within the health system and with other sectors:

I was shocked when I started working in primary health care with the number of patients taking benzodiazepines. There are a lot of people with medication prescribed by other colleagues from other units. Removing this medication took me a lot of time. In fact, patients didn't even want to go through consultation. They would come just for a new prescription. And it's been a very difficult job, but I try not to prescribe medication unless necessary. The patient is often placed in a therapeutic group. When he is involved with other activities, the emotional pain he feels attenuates. (Camila, family health physician)

I had an unemployed patient, with a problem at home and a mild depressive condition. I thought it was appropriate, at that moment, to put her on medication, but I don't think this is what will solve her problem. (...) To end up treating an issue that, from my point of view, is social with medication, I don't think this is the best way. When it is a physical illness, a bacterium, something like that, when you give the medication and it will resolve, the problem will be solved. But now the disorder is depressive because of... I believe... consultation times are reduced, and we can't address everything, but I believe it was because of socioeconomic difficulties at home. And I asked myself: how do I treat that with medication? I am not treating the cause, I think it will help, it will improve her mood, but it will not be the solution to the problem. (Bernardo, general practitioner)

Both narratives reveal a concern about the best approach to mental suffering. In the first one, we notice the abusive use of anxiolytics relates to the patient 's poor assessment in Camila's view. It also illustrates the difficulty in managing benzodiazepine prescriptions initiated by doctors from other healthcare units and the challenge of coordinating care by reconciling perspectives and interventions from different health teams, even if they work in the same territory. Bernardo in turn perceives something other than changes in mood in the patient 's behavior, but he feels helpless and chooses to prescribe psychotropic drugs for her. Camila’s and Bernardo’s concerns reflect both the responsibility for comprehensive care advocated in primary health care and the risk of medicalization. Interventions are shaped through structural failures in addressing health care in an intersectoral way. Moreover, such interventions might also end up conveying psychiatric or psychologized ways of understanding and facing suffering to the community (Menéndez, 2003).

Hence, social suffering ends up being integrated by the health system and health professionals treat mental suffering resulting from hunger, unemployment, and structural violence. The integration of social suffering by health teams reveals two opposites, but not exclusive, aspects of health care delivery in Brazil. On the one hand, a tendency of health services to take responsibility for not just health but also socioeconomic problems of the communities where they work. Probably, it is related to the influence of social determinants of health on the collective health movement and a broader view of health and illness that includes social and structural aspects (Ortega & Muller, 2022; Ortega & Wenceslau, 2020). On the other hand, they may end up producing unintended medicalization processes by restricting interventions to medication and psychosocial care because they cannot tackle social and economic inequalities with more straightforward social and economic interventions. Fragmentation between healthcare and other sectors, such as social care, brings about overload to primary health care, and shapes the view of illness and the interventions of health professionals and users. This lack of coordination between different sectors is not only evident in our fieldwork; it is a limitation of Brazil's public policies in local contexts and constitutes a structural determinant of healthcare. Work processes, health network management and sociocultural features of territory configure a social apparatus of attention and illustrate how the economy of attention provides a critical perspective on the impact of those contingent interactions influencing the recognition of health problems and the elaboration of their responses (Adams et al., 2019).

Our interviewees highlighted the existence of some resources available in the health system—prescription of (psychotropic) medication, complementary exams and referral to a mental health professional through the appointment regulation system—and we observed that there are different ways of dealing with these resources, as the following statements indicate:

I discussed a case at the mental health collaborative care meeting today. The patient had already told me that she had not been taking CAPS medication because she was taking the medication that her neighbor, who was also undergoing treatment at CAPS, was taking. She didn't even know the name of the drugs. Because when she didn't feel well, the neighbor said “look, this medication is very good. It makes me calm (...) If the medication works for me, why not use it? You don't have to go to the doctor”. We tried to explain to her that it was not allowed to share her neighbor’s medication. It is very complicated because it is a cultural issue. They don't think there is any harm in it. Unfortunately, if you do not take advantage of the power you have to try to show that it is a regulated thing, I do not know what might happen: Black market? In a little while they will be selling the medication (laughs). (Beatriz, general practitioner)

Many times, they [users] come here with this idea that we are a referral center (...). They think that entry into the outpatient care system only happens through primary health care. And that any demand can be included in the system. (Bernardo, general practitioner)

Primary health care is the level of care within the health system responsible for entering the health care network. So, patients are aware of this information and practitioners assess the need for referral for specialist assessment. What was noticed in the interviews is that doctors with more experience in family health units and more knowledge of the territory's resources seemed to deal with this demand in a more routine way. In unit B, where a higher turnover of professionals was observed, the request for external referrals may constitute a pragmatic strategy of patients regarding their own care, in order to ensure evaluation and follow-up, even if there are difficulties to be attended by the unit’s physician (Müller, 2019).

Therefore, those interventions—be they prescriptions, examination requests or referrals—should be proposed considering they have a *social life* as soon as they are prescribed. Hence, patients and their micro-social groups articulate health strategies on their own in a struggle to take care of themselves. Individual and social groups promote articulations between care interventions, which also reflects a pragmatic stance towards their afflictions (Menéndez, 2003, 2016; Müller et al., 2022). From the perspective of the economy of attention the arrangements that individuals and their social micro-groups make to care for themselves should be identified by health professionals as an expression of the intertwining between health phenomena and the social dimension. Those arrangements constitute strategies to cope with mental distress and structural vulnerabilities and reflect the availability of resources in the community and the broader structural limits engendered by the disarticulation of public assistance policies (health, education, social security, leisure, urban planning).

An important aspect to be stressed in this context is the role played by community health workers within Brazilian mental health collaborative care. Community health workers welcome new patients, make home visits to follow-up families, and may participate in group therapeutic activities at health units. In addition to knowing the territory where health services are located, they also share local history and values with families, mediating the relationship with health professionals and services (Nunes et al., 2002, 2007). Due to these characteristics, the insertion of community health workers in the teams is highly important. Even so, the interaction with other health teams professionals might be troublesome, as evinced in the following excerpts:

The community health worker would judge her because she had known them for a long time and she would say “no, I'm not going to talk to her because I know their kind.” Her father killed a lot of people. Because he has a history, because the community health worker has lived there since she was a kid and knows what the patient’s father did or didn't do. This is very crazy. Sometimes they are afraid because they know us, but we [Family health professionals] are not there as a gossiping neighbor. We are there to play another role. Yes, it is difficult to separate these relations. Sometimes I think that there is no way out of it. (Elisa, mental health professional).

The issue of community health workers’ participation is super complex because there is a huge demand in relation to their goals. When the guys [CHW] start participating in the groups, they like it, they have several ideas. We can do a great job. (André, mental health professional).

In the first excerpt, a mental health professional describes the community health worker's refusal in treating a family due to her prejudice towards the father, a known drug dealer and murderer. The mental health professional argues that their job is to take care of people no matter their background. However, the community health worker might not spend enough time on their visits or fail to look for alternatives in care for this family.

The difficulty for the CHW to carry out home visits observed by Elisa was associated with prejudice against the patient's father. However, the issue of the risk of violence for the CHW was not even mentioned, nor was the history of violence related to drug trafficking in that community and its effects on social interactions. This detachment from the social reality of some health professionals expresses a challenge for health training (Ortega and Müller, 2022), but an even greater challenge for socially and culturally sensitive practices (Goulart et al, 2021).

The second excerpt shows another face of the family health strategy. All health professionals, including community health workers, must deal with a specific workload, so they must visit and serve a certain number of families monthly, reducing group interventions, as well as the opportunities to include CHW's experiences in team meetings more systematically. The advance of neoliberal policies in recent years has triggered some changes in primary health care guidelines with direct effects on team formation and care management (Portaria No. 2.436, 2017; Morosini & Fonseca, 2018). The restriction of CHWs’ participation in the interventions developed by the teams is one example of this restructuring of PHC. It represents an important setback for the development of quality care practices and reflects the “systematic relegation of integrated community-based approaches” (Almeida, 2019, p. 4).

## Discussing Mental Health and Family Health Within the Economy of attention framework

Our results evince that primary health care and mental health professionals promote different strategies and therapeutic negotiations to identify and address mental suffering. In the Brazilian context, specifically in Rio de Janeiro, those strategies are related to the interweaving of three social dimensions involved in the provision of health care: team composition and organization of work processes; sociocultural characteristics of the territory and availability of resources in the community; and the broader sociopolitical structure of the

SUS. Therapeutic negotiations varied according to the level of sensitivity to the social processes at play in each clinical encounter.

Regarding professional training, work experience at primary health care and the type of therapeutic activities that are developed were decisive for framing patients' complaints, or presentations, in terms of their need for mental health interventions. Thus, physicians with less experience in family medicine used to think more in terms of conventional psychiatric classifications, while community health workers, mental health professionals and physicians with longer working experience at primary health care draw on broader frameworks regarding mental suffering, socioeconomic status, cultural values and relational family and neighborhood dynamics (Pinto, 2017). Work processes that favor individual consultation over group therapy, or that restrict the participation of community health agents, as pointed out by some interviewees, increase the risk of normalizing habits, knowledge, and values, restraining the possibilities of integrating the sociocultural characteristics of the communities and therapeutic negotiations (Müller, 2019). Even if active listening or person-centered anamnesis are useful tools for sensitive care, those approaches need to be planned and nurtured as managerial and political decisions along with other strategies, such as home visits and community resources mapping (Santos et al., 2017). If work processes are very strictly planned, they may limit more inclusive interventions.

Socioeconomic features in the territory also influenced physicians and case management and discussions with mental health professionals. They recognized that those factors impact on suffering expressions and help seeking behaviors, which may be related to the presence of health services in their catchment area (Ortega & Müller, 2022). Regarding large urban centers, such as Rio de Janeiro, health professionals have direct contact with the urban and sociocultural diversity in the region. Thus, contact with the violence resulting from drug trafficking, police incursions and paramilitary control, or else, due to the difficulty of urban access, insufficient leisure areas and schools, transform the possibilities of understanding and intervening in the health-disease phenomenon (Goulart et al., 2021).

The reports we presented showed the efforts and sensitivity of professionals in implementing appropriate solutions along with social actors. However, tensions were not always negotiated under fair conditions. Either patients had to yell to make themselves understood, or prescriptions (for psychotropic drugs) had to be kept under lock and key in order not to be “trafficked”, or “conniving mothers” with their sons’ choices (of not to be treated) had to be sought and challenged. When it comes to case management difficulties in mental health services, refusals and abandonments are commonly assessed in a reductionist way and social, cultural and political dimensions are neglected (Müller et al., 2022), impoverishing clinical experience and, mainly, accentuating unequal relationships between health professionals and patients (Farmer et al., 2006).

The non-adherence, or disagreement, to mental health treatments is an illustration of this tendency. Alice and Beatriz patients' disagreement with the diagnosis or prescription were interpreted as an inability to understand the medical vocabulary, due to less education or a prejudiced attitude towards mental illness. These readings of the difficulties of the doctor-patient relationship reflect a static understanding of social phenomena, as well as a delegitimization of patients' narratives. Thus, non-adherence to treatment may evince access barriers due to treatment disagreements, which could not be accepted or negotiated, and engender a cycle of structural exclusion and violence (Farmer et al., 2006). What those professionals overlook is that users’ and their families’ disagreements with treatments involve complex negotiations and socioeconomic, territorial and technical structures underlying their actions. Therefore, users’ care practices and therapeutic itineraries evince wider social and political determinations. They deploy a pragmatic attitude towards care practices and treatments to ensure the biosocial reproduction process (Menéndez, 2003, 2016).

As we mentioned previously, sensitivity to the sociocultural elements of the health-disease phenomenon allows professionals to deepen approaches and diversify interventions. Furthermore, the recognition of users as agents of care practices includes them in the construction of care alternatives, enriching therapeutic negotiation.

Notably, training in family and community medicine in Brazil is highly praised in advancing good practices in primary health care, guided by cultural sensitivity and community orientation (Schneider et al., 2020). However, the tensions we see in the interviews with our informants may be linked to particularities of the health training provided at medical schools. While such training involves knowledge of social and cultural determinants of health, it also embodies a limited understanding of the historical-social context and of lay practices that express communities’ social imaginary related to the health-disease-seeking care phenomena (Müller, 2019).

Adopting the perspective of therapeutic negotiation is useful for primary health care settings, but also for developing participatory care projects, sensitives to local complexities, in dialog with broader contexts (Bemme, 2019). Thereby, health professionals ought to understand themselves and users as embedded agents in a more dynamic social context (Adams et al., 2019; Chase, 2021).

Finally, the question of the political-administrative organization of the Brazilian health system is also a bottleneck for mental health care at primary health care (Bousquat et al., 2019; Salazar et al., 2017). SUS is guided by PHC and the health care network is set in a hierarchical and regionalized way, in which the gateway to the system and organization of the service flow are managed by the family health network. The development of care policies is based on an expanded conception of health, critical of biomedical reductionism and that seeks to develop interventions and care devices guided by comprehensiveness, understood as the articulation of promotion, prevention and health care, but also as the coordination of care at different care levels within the health services network (Paim et al., 2011). MHCC in Brazil is an expression of these values because it constitutes a collaborative care strategy aimed at learning in services and through dialogical interaction between health professionals with different knowledge, planning therapeutic interventions together, and negotiating care flows in the health care network (Ortega & Müller, 2022).

Still, there are many hurdles for the system to run properly. Not all municipalities are able to guarantee quality assistance coverage to the population. They struggle in key points as financing, managing the responsibilities of each level of care, communication between teams and services and intersectoral interventions. Furthermore, there is an effort in training family and community health professionals and retaining them in the services (Almeida, 2018; Santos et al., 2017). Among the interviewees in this study, the excess of service demands associated with unpredictability regarding hiring time and salaries will likely influence the decision of leaving the unit, reducing capacity building opportunities and impacting on the quality of care (Faregh et al., 2019).

Professionals noticed hurdles in work processes, fragmented health service networks, resourceless territories in which they acted in different contexts. Moreover, those systemic failures seemed to be naturalized and not related to limitations of Brazil's public policies coordination between healthcare and other sectors, such as social care in local contexts. In this regard, the turnover of professionals in family health teams, especially physicians, should not be taken as fortuitous. It impacts users’ and professionals’ relationships. Units with higher rates of physician mobility showed more requests for referral to other levels of care and more reports of self-medication and therapeutic negotiation difficulties. Likewise, limitation of care activities carried out by community health workers and its concentration within the health units as a result of recent changes in PHC policies, will potentially reduce the effect of their intermediation with the community (Morosini & Fonseca, 2018).

The “economy of attention” framework helped to interpret diagnostic and therapeutic approaches, as evinced in the interviews we conducted for our research. Although some professionals acknowledged the role of social determination in mental suffering, they had little to do besides prescribing medications. This was especially true among lesser experienced physicians. On the other hand, broader solutions were achieved whenever users’ interactions and demands were considered by family health teams. One can infer that health interventions sensitive to complex interweaving of material and human conditions may avoid the reductionism engendered by the ignorance of complex social causality (Adams et al., 2019; Lie & Greene, 2021).

Values and discourses advanced by health professionals, researchers, and managers are guided by criticism of biomedicine. Much of what is planned comes close to the idea of sensitivity care, but the fragmentation of the system and the difficulty in training and retaining professionals reflects these frictions that professionals experience in their everyday lives. Family health strategy is effective, powerful, and so is the mental health collaborative care, but it is difficult to implement because of all the clashes and “de-financing” that define the Brazilian scenario (Ortega & Orsini, 2020; Ortega & Müller, 2022).

Although user advocacy movements increasingly influence health planning and public policies, including these agendas in public debate requires a nuanced understanding of the sociocultural dimension (Adams et al., 2019; Ortega & Müller, 2021). Users and their social micro-groups develop healthcare strategies drawing on their individual and community health and illness conceptions, affirmation of identities, establishment of alliances and articulation with different resources in and outside the health field. Those interactions may be useful to reflect upon strategies of social action to access health resources. Even if not systematized in concrete forms of activism, they mirror social networks and structural conditions in which users and health care professionals are embedded (Ortega & Müller, 2021).

## Conclusions

In this article, we discussed the identification and management of mental health cases by health professionals in primary health care in the city of Rio de Janeiro. Professional-patient relationships, the characteristics of health services and other structural aspects have generated unique therapeutic negotiations. An expanded understanding of the health-disease-seeking-for-care phenomenon focused on community health services, as endorsed by SUS, has stimulated the recognition of sociocultural elements in the reported situations. However, tensions and neglects around diagnostic procedures and healthcare interventions were seen among interviewees. We argue that healthcare work processes, community features and health system bottlenecks are part of any social care apparatus. Moreover, the tendency to plan interventions focused on unique problems, or else, without integration amidst different sectors, ends up disregarding complex social conditions, which would reinforce fragmentation and reduce the impact of those interventions (Adams et al., 2019; Faregh et al., 2019).

It is an important task in mental health care to recognize the indefinite, borderline character of these “psychosocial problems”, taking the investigation of how they are produced in the daily care practices (Chase, 2021). Such a task will contribute to the construction of more proper interventions locally tailored, but it will also assist in the process of translation into other contexts. Translation understood as a search for cooperation between the agents that make up the field of mental health care (Chase, 2021).

It is in the everyday interactions that the processes of incorporating the different epistemologies and logics of care are manifested. Focusing on negotiations enables appreciation of the role played by social actors in these exchanges and other features related to access and quality of care (Menéndez, 2016). Finally, it allows us to develop interactive and dynamic care strategies that are so necessary for objects as open and contested as mental health care.
